# Systematic Review on the Potential Effect of Berry Intake in the Cognitive Functions of Healthy People

**DOI:** 10.3390/nu14142977

**Published:** 2022-07-20

**Authors:** Ramona De Amicis, Sara Paola Mambrini, Marta Pellizzari, Andrea Foppiani, Simona Bertoli, Alberto Battezzati, Alessandro Leone

**Affiliations:** 1 International Center for the Assessment of Nutritional Status (ICANS), Department of Food, Environmental and Nutritional Sciences (DeFENS), University of Milan, 20133 Milan, Italy; sara.mambrini@unimi.it (S.P.M.); marta.pellizzari@unimi.it (M.P.); andrea.foppiani@unimi.it (A.F.); simona.bertoli@unimi.it (S.B.); alberto.battezzati@unimi.it (A.B.); alessandro.leone1@unimi.it (A.L.); 2 Laboratory of Nutrition and Obesity Research, Department of Endocrine and Metabolic Diseases, IRCCS, Istituto Auxologico Italiano, 20145 Milan, Italy; 3 Laboratory of Metabolic Research, S. Giuseppe Hospital, IRCCS, Istituto Auxologico Italiano, 28824 Piancavallo, Italy

**Keywords:** berries, cognition, human intervention studies, systematic review

## Abstract

The increase in life expectancy poses health challenges, such as increasing the impairment of cognitive functions. Berries show a neuroprotective effect thanks to flavonoids, able to reduce neuroinflammatory and to increase neuronal connections. The aim of this systematic review is to explore the impact of berries supplementation on cognitive function in healthy adults and the elderly. Twelve studies were included for a total of 399 participants, aged 18–81 years (mean age: 41.8 ± 4.7 years). Six studies involved young adults (23.9 ± 3.7 years), and four studies involved the elderly (60.6 ± 6.4 years). Most studies investigated effects of a single berry product, but one used a mixture of 4 berries. Non-significant differences were detected across cognition domains and methodologies, but significant and positive effects were found for all cognitive domains (attention and concentration, executive functioning, memory, motor skills and construction, and processing speed), and in most cases they were present in more than one study and detected using different methodologies. Although some limitations should be taken into account to explain these results, the positive findings across studies and methodologies elicit further studies on this topic, to endorse the consumption of berries in healthy populations to prevent cognitive decline.

## 1. Introduction

Cognitive function refers to a variety of mental abilities such as perception, attention, memory, decision-making, and language comprehension. It changes over one’s lifetime, improving from childhood to young adulthood and showing a gradual decline in elderly [1]. The increase in average life expectancy poses health challenges such as increasing the impairment of cognitive functions with an estimated global prevalence ranging from 12% for subjective cognitive decline to 18% for dementia, increasing with the growing age and without gender differences [2,3]. Cognitive impairment is a physiological process that leads to a deterioration in the cognitive abilities of the individual, significantly interfering with everyday life. Its spectrum in adults is very broad, ranging from the physiological decline associated with ageing, through various states of cognitive decline with memory impairment to dementia [4]. To date, there are no treatments for these diseases; however, this process can be exacerbated or aggravated by the presence of risk factors such as smoking, being overweight and obesity, cardiovascular problems and lifestyle issues such as diet [5,6]. Therefore, there is a growing interest in new lifestyle strategies that can prevent or delay the onset of neurodegeneration: diet in particular can play a neuroprotective role, and can potentially be a well-tolerated, inexpensive, and effective alternative to protect against age-related cognitive decline and neurodegeneration, resulting in significant personal and social benefits [7].

In 2015, a specific diet for the prevention of cognitive impairment was developed: the MIND diet. This is a Mediterranean-style diet characterized by the addition of foods rich in bioactive compounds useful for the neuronal mechanisms contained in specific foods. The Mediterranean-Dietary Approaches to Stop Hypertension (DASH) for Neurodegenerative Delay (MIND) diet is a hybrid of the Mediterranean and DASH diets with selected modifications based on the most compelling evidence in the diet-dementia field. The MIND diet features the consumption of vegetables (particularly, green leafy vegetables), berries, extra-virgin olive oil, nuts, whole grains, and low-fat sources of protein [8,9,10]. Concerning berries, their neuroprotective actions appear to be due to a group of molecules called flavonoids, and in particular anthocyanins, contained in many berries. The neuroprotective effect of flavonoids is well documented in the literature, particularly in vitro and in animal models [11]. Their ability to block the propagation of oxygen-free radicals (ROS), and at the same time to pass through the blood-brain barrier, makes them able to reduce the neuroinflammatory state often behind neurodegenerative diseases [12]. Flavonoids are able to interact with neuronal structures responsible for information storage and memory. They have demonstrated the ability to increase and strengthen neuronal connections and to increase growth factors, such as Brain-Derived Neurotrophic Factor (BDNF), that regulate the development and maintenance of neuronal functions, resulting in regeneration of brain cells and neurogenesis. Flavonoids have also been shown to improve and increase blood flow in the brain [13], increasing vascularization and angiogenesis in all areas of the brain, such as the memory hippocampus [14].

Translating this evidence obtained in vitro or in animal models is not as easy as in human clinical studies. First, bioactive components undergo absorption and distribution kinetics linked to human metabolism, which can therefore modify their bioavailability. In addition, these bioactive components are included in a food matrix with which they interact synergistically. An approach that aims to prevent certain diseases through nutrition cannot be based exclusively on the concept of the nutrient (e.g., anthocyanins, flavonoids), as this would simplify the complexity of the food matrix and lose the central concept of the dietary pattern. We use the term food synergy to denote this health action of the food matrix [15,16,17]. Thus, to understand the true role of berries in protection on cognitive function we should consider in vivo studies that consider the dietary pattern in general. Some recent reviews have reported as the synergistic biochemical interactions among nutrients found in berries exert a multiplicity of neuroprotective actions, especially when consumed in the MIND dietary pattern that was moderately associated with better verbal memory in later life or a decrease in Alzheimer’s disease risk [7]. They have also shown how dietary supplementation with berry fruit can alter cognitive performance, perhaps forestalling or reversing the effects of neurodegeneration in aging, and can delay cognitive aging by up to 2.5 years in women >70 years of age [18]. Nevertheless, the association between berry consumption and cognitive function remains limited and equivocal.

Therefore, the aim of this systematic review is to synthesize the current available literature by further exploring the impact of berries supplementation on cognitive function in healthy adults and the elderly.

## 2. Materials and Methods

### 2.1. Search Strategy

This systematic review has been performed according to the Preferred Reporting Items for Systematic Reviews and Meta-Analyses (PRISMA) guidelines [19]. The research question addressed whether berry consumption influences cognitive function on adults and/or elderly without cognitive impairment. Keyword selection was based on the PICO(S) framework (Population, Intervention, Comparison, Outcome, Study Type) [20]. We conducted a naive search in the Pubmed, Scopus, and Web of Science e-databases, selecting articles published until March 2022. To reduce bias in search strategy development, we used text mining on titles and abstracts of the deduplicated records captured from the naive search to build a keyword co-occurrence network able to identify recurrent synonymous terms omitted in the naive search [21].

The search string augmented by automatic identification of search term was: ‘((“extract* combin*” OR berr*) AND (“blood* pressur*” OR cognit* OR attent* OR memori* OR languag* OR mental* OR visuospati* OR execut*) AND (adult* OR elder*) AND (“blind* design*” OR “control* doubl*” OR “control* trial*” OR “doubl* blind*” OR “placebo* control*” OR “random* control*” OR rct))’.

### 2.2. Eligibility Criteria

Studies were included if they: evaluated the consumption of berries (regardless of the form) in adults and elderly without cognitive impairment (including mild cognitive impairment (MCI)) (1); used a randomized controlled trial (RCT) study design (2); were published in English (3); and the outcome was related to cognitive function (4). No systematic reviews or meta-analyses were included in this study.

### 2.3. Study Selection

Right after excluding all duplicates, two reviewers evaluated the title and abstract of the articles identified through the database searches independently. Once selected the relevant articles, they analyzed further following the eligibility criteria. Afterward, the full texts of the included articles were reviewed for the final inclusion. When the two reviewers disagreed, a third reviewer was involved, and resolved any discrepancies by making the final decision.

### 2.4. Data Extraction

Papers were reviewed to obtain the following information: year of publication, authors, country, study design, description of the study population (sample size, age, sex, comorbidities or health condition), characteristics of the berry used in the intervention (genus or species, type of preparation, other bioactive compounds), posology (daily doses, frequency, timing, duration), control product administered, details about the outcomes (parameters collected, tools and results), confounders used to adjust the analysis, and intervention effects.

### 2.5. Quality Assessment

We assessed risk of bias in the included RCTs using the Revised Cochrane risk-of-bias tool for randomized trials (RoB 2) [22]. The tool is structured into five domains through which bias might be introduced into the result: (1) bias arising from the randomization process; (2) bias due to deviations from intended interventions; (3) bias due to missing outcome data; (4) bias in measurement of the outcome; and (5) bias in selection of the reported result.

## 3. Results

### 3.1. Study Selection

**Figure 1:**
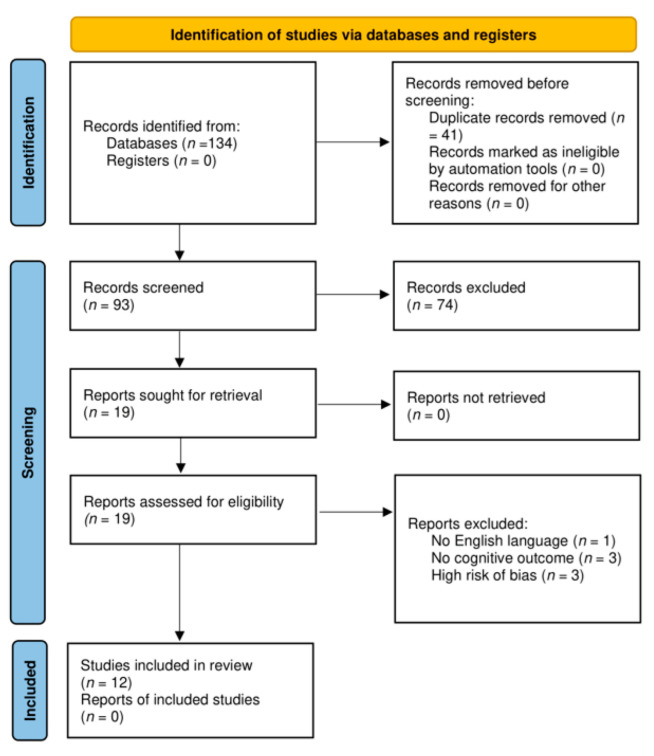
Flow-chart of the selected studies.

The flow-chart of the literature search and exclusion process is shown in Figure 1.

We initially retrieved 134 potential articles published until March 2022. After excluding 41 duplicates, we retained 93 articles. Next, we discarded 74 irrelevant articles based on title or abstract (31 did not report a cognitive outcome, 28 were excluded as they were not RCT, 12 did not use berries of berry preparation as intervention, and 3 were performed in children or adolescents). The remaining 19 records were assessed for eligibility. We discarded 1 article not written in English and 3 studies not in line with the purpose of this review. A complete report of study characteristics and RoB 2 results are available in Table S1 of the Supplementary Materials. Studies that had as overall a low risk of bias (10 studies) or some concerns (2 studies) were considered acceptable for inclusion in the systematic review, conversely studies that had high risk of bias (3 studies) [23,24,25], mainly due to the randomization process, were excluded (see Figures S1 and S2 of the Supplementary Materials). Finally, 12 studies were included for the present review [26,27,28,29,30,31,32,33,34,35,36,37].

### 3.2. Selected Studies Characteristics

The characteristics of selected studies are summarized in Table 1. The 12 studies [26,27,28,29,30,31,32,33,34,35,36,37] included 399 adults. The sample size ranged from 9 to 40 individuals receiving the intervention, aged 18 to 81 years (mean age: 41.8 ± 4.7 years). Six studies involved young adults (23.9 ± 3.7 years) and four studies involved the elderly (60.6 ± 6.4 years). Two studies included only females, other studies included more than 80% females, while in the remaining studies sexes were more evenly represented (2 studies did not report sex distribution). One study was performed in New Zealand and one in the US, all other studies were conducted in Europe. All included studies were RCTs: five were parallel RCT and seven used crossover design.

A total of 5 cognitive domains were investigated in selected studies: attention and concentration; executive functioning; memory; motor skills and construction; and processing speed. Most studies studied more than one domain, but great heterogeneity between studies was found in parameters and methods used to assess a specific domain.

Most studies investigated effects of a single berry product, but one used a berry mixture of 4 berries. Crataegus berries and blueberry were used in 3 studies each, while other berries were used in the remaining studies. Preparation of the studied berry varied, and included prevalently extracts (7 studies, mostly from commercial preparations), standardized juices, and powdered preparations. Most studies investigated acute effects, although 2 studies investigated daily administrations of berries for periods of 12 and 24 weeks.

### 3.3. Effects on Cognition

In most cases, non-significant differences were detected across cognition domains and methodologies, but significant and positive effects were found for several domains of cognition, and in most cases, they were present in more than one study and detected using different methodologies.

The amount of bioactive compounds (anthocyanins or other polyphenols) investigated in all studies ranged from 16 mg in the extract of Aronia melanocarpa [28] to 725 mg of polyphenols in the wild blueberry [24].

Attention and concentration was evaluated in 9 studies [26,28,31,32,33,34,35,36,37] by the Alertness Test, Digit Symbol Test, Digit Vigilance Task, Stroop Task, Bond-Lader Alert, Attention Network Task, CogTrack™, Continuous Performance Task, Commission Errors, Number Cross-Out Test and Attention Summary. Improvements were detected in 3/9 studies [32,33,34] and with 2 different methodologies. Improvements were related both to reduced reaction time and reduction in the number of errors. Two studies evaluated the effect due to prolonged exposure (12 and 24 weeks), but did not detect a significant effect.

Executive function was evaluated in 5 studies [24,29,33,35,37] by Modified Attention Network Task, COMPASS, Go-NoGo, Correct Reaction Time, Digit Symbol Substitution Test, Total Correct, Serial Subtraction, 3s and 7s, and Logical Reasoning. Improvements were detected in 1/5 studies [35] and with 3 different methodologies. Improvements were related to response time and number of errors.

Memory was evaluated in 5 studies [24,31,34,35,37] by Immediate Word Recall, Auditory Verbal Learning Task, Random Word Generation, Total Correct, Three-Word Sets, Total Correct, Memory Summary, and Working Memory Summary. Improvements were detected in 2/5 studies [30,35] and with 1 methodology. Improvements were related to accuracy in recognition or rejection during recollection.

Processing speed was evaluated in 9 studies [26,27,28,29,31,32,33,36,37] by Processing Speed and Attention Summary, COMPASS, Stroop Color and Word Test, Stroop, Correct Reaction Time, Digit Switch, Switch Cost, CogTrack™, Rapid Visual Information Processing (RVIP), Number-Connection-Test, Digit-Symbol-Test, Wechsler Adult Intelligence Scale, and Attentional Performance Test – ‘‘Test d2”. Improvements were detected in 4/9 studies [26,27,33,36] and with 4 different methodologies.

Motor skills and construction were evaluated in only one study [28] by the Grooved pegboard test, which detected improvements with one methodology. Improvements were related to manipulative dexterity. Improvements were detected after prolonged exposure (24 weeks).

Several studies also assessed biological parameters related to cognition:

− 6 studies evaluated blood pressure [26,27,28,32,35,37], showing an increased diastolic blood pressure in 4 studies [26,27,32,37] and lower diastolic blood pressure in 2 studies [28,35];
− one study [33] evaluated the platelet monoamine oxidase A and B (MAO-A and -B) activity in healthy young humans, observing a clinically significant inhibition of platelet MAO-B following blackcurrant supplementation;
− one study evaluated modulation of pre-frontal cortex brain wave spectral activity measured by electroencephalogram (EEG) reporting an anxiolytic effect following blackcurrant juice drink somministration;
− 3 studies [28,31,37] evaluated BDNF levels, and one nerve growth factor receptor (NGF-R) [31], detected related improvements in some cognition domains in 1/3 studies [37];
− one study [31] assessed the total polyphenols levels in urine, failing to find significant difference in neurotrofin levels (BDNF and NGF-R), but showing an improvement in executive function; and
− one study [29] evaluated also mood and the cerebral blood flow with three different active beverages reporting an increased subjective energetic arousal and hemodynamic responses.

## 4. Discussion

Overall, our review of the literature regarding the effect of berries consumption on cognitive function in healthy subjects was unable to find consistent effects across studies and methodologies, as for the majority of outcomes, no significant effects were found. On the other hand, for most cognitive domains that were assessed, more than one study reported a significant and positive effect assessed with different methodologies.

Current evidence [38] suggests that the positive effect of berry consumption, both acute and chronic, is due to their content of vitamin C and phytochemicals, in particular anthocyanins. They also contain ellagitannins, flavan-3-ols, procyanidins, flavonols and hydroxybenzoate derivatives [38]. These bioactive compounds are, in fact, strongly involved in reducing oxidative stress and inflammation involved in cognitive behavioral deficits [39], replacing endogenous antioxidants that naturally decline in aging and reducing the brain’s vulnerability to the deleterious effects of oxidative damage [39]. These typical berry compounds could act at different levels influencing cognition: i.e., they can act on both hypertension and hypotension, influencing cerebral perfusion and improving cognitive performance [40]; they affect neurotrofin expression (as BDNF and/or NGF-R), associated with both normal and pathological ageing, particularly in areas important for memory processes [41]; furthermore, anthocyanins have been shown to have inhibitory effects on MAO-A and -B, producers of hydrogen peroxide, thus reducing the oxidative stress associated with this process and leading to increased concentrations of these monoamine neurotransmitters, essential for normal cognitive function and mood [33,42].

However, these effects in humans are dependent on the bioavailability of these compounds, i.e., the integrity of the main sites of absorption and catabolism of these components and their interaction with the colonic microflora, demonstrating how berry consumption can modify the colonic environment and how a specific microbiota can modify the reaction towards berry absorption, catabolism and bioactivity in the brain [38]. This aspect, together with the different studied populations, the multiplicity of study design, the small sample size, and the variety in type and daily doses of berries, could explain the modest positive effects in our review.

First of all, most studies are conducted on very similar samples, making it difficult to generalize the results: mostly women, from Anglo-Saxon countries and young people, in which cognitive functions, such as memory, concentration, problem solving, comprehension, are most likely directly associated with real benefits, but are difficult to identify. By contrast, the elderly population, who is more at risk of cognitive impairments but studied in only 2 of the RCTs included, could show greater benefits with the same intervention reversing the effects of neurodegeneration in aging [43].

In addition, this review shows how the evaluation of cognitive function poses several challenges. In most studies investigating the state of cognitive improvement, structured interviews, tests or questionnaires are used, which are indirect methods of assessing the state of cognition; in all the studies reviewed for this paper, cognitive domains were assessed indirectly, nine studies [26,27,28,29,31,32,33,35,37] also included an evaluation of a biological parameter expressing brain function: although two studies did not report improvements in cognition after administration of berries, probably due to the short duration of the study, they did report an improvement in associated biological parameters (e.g., Blood pressure or BDNF levels). Moreover, it is well known that cognitive test performance may improve with repeated assessments, a phenomenon referred to as procedural learning or practice effects. On the other hand, cognitive tests are characterized by brevity, portability and simplicity, and are surely non-demanding for the client, also allowing a tailored approach to treatment; however, their brevity generally compromises reliability, not all domains are investigated, and they are not sensitive enough to detect impairment in high functioning and/or well-educated people, as they are strongly influenced by education, literacy, and cultural or linguistic background. Moreover, large interindividual differences in responsiveness hampers a clear substantiation of the potential health effects of nutrients in intervention studies [43]. Another fundamental consideration concerns the protocols used to assess cognitive function. It is possible that protocols that examine variables of cognitive function only once do not show any changes, whereas studies that expose participants to repeated tests or a longer protocol that induces cognitive fatigue do show a difference. Thus, polyphenol changes on cognitive function may appear when exposed to longer tests compared to a short test protocol.

To date, efforts are being made to identify biological markers that can be used as a direct marker of cognitive impairment, even in very early stages. High levels of interleukin-10 (IL-10), interleukin-1 (IL-1), Tumor Necrosis Factor (TNF)-alpha have been observed in patients with cognitive impairment. Certain vascular endothelial growth factors (VEGF) were also found to be positively related to cognitive impairment. This marker could offer unique advantages for implementing preventive programs on a large scale, and will allow the identification of preclinical neurocognitive disorders in the community [44,45]. Measuring the effects of nutritional intervention directly on brain structure, composition, and activation using various brain imaging techniques can be valuable. Although such findings are not readily translated into functional (behavioral) benefits, they may provide highly sensitive measures of nutritional effects.

Other methodological factors may lie behind the lack of a positive association: eleven studies among those selected considered habitual polyphenol intake or overall dietary pattern or standardized diet prior to testing [26,27,30,31,32,33,34,35,36]. Greater emphasis should be posed on the overall diet of participants while trying to detect the effect of the consumption of a single food. The evaluation of dietary patterns avoids potential confounding with berries consumption, making it possible to assess the concomitant consumption of food sources of polyphenols, such as those contained in berries, increases the ability to evaluate stronger effects due to the cumulative effects of many dietary characteristics, and allows for the evaluation of the interaction between synergistic components [17,46,47]. Indeed, foods are complex combinations of nutrients and other compounds that act synergistically within and between food combinations [17]: berry consumption in a varied diet that is balanced in terms of calories and nutrients may not have the same effect when consumed in a dietary pattern that is high in calories [47]. So, in order to assess the effect of berry consumption on cognitive outcomes, it is also important to evaluate the habitual consumption of products containing polyphenols in the diet that could have a synergistic effect with those of berries. This very important aspect could be assessed by means of a recall-24 or food diary to collect the subjects’ eating habits.

Another important observation concerns the type and the form of administration of the active substance. Different berries may act differently to produce their positive effects [39]. The composition and content of bioactive compounds in berries vary depending on the cultivar and variety, the place of cultivation and environmental conditions, the nutrition of the plant, the stage of ripening and the time of harvest, as well as subsequent storage conditions or processing methods [48]. This different composition, as opposed to drugs, makes it difficult to titrate the dosage of the product to obtain a supplement dose that allows maximum benefit without adverse effects, and to translate supplementation into an effect. Indeed, it seems known that higher doses of polyphenols (>500 mg/day) may be required to attenuate cognitive decline, while only smaller daily doses are likely to be effective [49]. Thus, as raw berries have different polyphenol contents, different types of administration are required to achieve an effect. For example, berries with a higher polyphenol content can be administered as fresh juice, while those with a lower polyphenol content should be processed into an extract in order to be effective.

Nevertheless, when detected, the impact of berries consumption on cognitive function was positive, with only one exception [36]. The authors argued that the unexpected result (i.e., reduction of processing speed following berries consumption) could be due to enhanced or deep relaxation caused by the anxiolytic effects of the anthocyanin-rich blackcurrant juice.

## 5. Conclusions

In conclusion, the selected studies had several limitations. Sample sizes were relatively small when considering both the inter-personal variability in cognition outcomes and the expected size of the effect due to berries consumption. While acute studies pose fewer challenges in study design and the assessment of cognitive function, the impact of chronic consumption should also be emphasized. Greater emphasis on confounders, especially dietary confounders, is also warranted. Although some limitations should be taken into account to explain the results of this systematic review, the positive findings across studies and methodologies elicit further studies on this topic to endorse the consumption of berries in healthy populations to prevent cognitive decline.

## Figures and Tables

**Table 1:** Characteristics of the selected studies [26,27,28,29,30,31,32,33,34,35,36,37].

Study	Study Design	Sample Size ^1^	Age (years) ^1,2^	Berry	Daily Dose	Intervention Duration	Outcome Domain	Effect
Schandry and Duschek (2008)	Parallel RCT	19 (21 controls)	25.8 ± 5.0	Crataegus berries	25 drops = 0.97 g berry extract ~ 24 g fresh fruit	Acute effect	Processing speed (Attentional Performance Test—‘‘Test d2′’)	Significant and positive effect as assessed by the Number-Connection-Test
							Attention and concentration (Alertness Test)	No significant differences found
		24 (24 controls)	25.9 ± 5.0	Crataegus berries	25 drops = 0.97 g berry extract ~ 24 g fresh fruit	Acute effect	Processing speed (Number-Connection-Test-Wechsler Adult Intelligence Scale, Digit Symbol Subtest)	Significant and positive effect as assessed by the Number-Connection-Test and the Wechsler Adult Intelligence Scale (Digit Symbol subtest)
Werner et al. (2009)	Parallel RCT	40 (40 controls)	59.25 ± 8	Crataegus berries	25 drops = 0.97 g berry extract ~ 24 g fresh fruit	Acute effect	Processing speed (Number-Connection-Test, Digit-Symbol-Test)	Significant and positive effect as assessed by the Wechsler Adult Intelligence Scale (Digit Symbol subtest)
Erfurt et al. (2014)	Parallel RCT	38 (15 controls)	24.4 ± 4.4	Crataegus berries	4 × 20 drops = 3.1 g berry extract ~ 78 g fresh fruit	Acute effect	Attention and concentration (Digit Symbol Test)	Significant and positive effect as assessed by the Test d2
							Processing speed (Attentional Performance Test—‘‘Test d2′’)	No significant differences found
Watson et al. (2015)	Cross-over RCT	36	24.8 ± 3.93, (18, 34)	Blackcurrant	1.66 g berry extract ~ 525 ± 5 mg of polyphenols (values per 60 kg of bodyweight) 142 mL ~ 150 g fresh fruit ~ 499 mg of polyphenols (values per 60 kg of bodyweight)	Acute effect Acute effect	Attention and concentration (Digit Vigilance Task)	Significant and positive effect as assessed by the COMPASS test (Digit vigilance reaction time)
							Processing speed (Rapid Visual Information Processing (RVIP))	Significant and positive effect as assessed by the COMPASS test (Rapid visual information processing accuracy)
							Executive functioning (Logical Reasoning)	No significant differences found
							Attention and concentration (Stroop Task)	No significant differences found
Haskell-Ramsay et al. (2016)	Cross-over RCT	20	21.05 ± 0.89	Purple grape	230 mL ~ 1681.7 μg/m of polyphenols	Acute effect	Memory (Immediate Word Recall)	No significant differences found
							Attention and concentration (Bond-Lader Alert)	Significant and positive effect as assessed by the COMPASS test (Attention reaction time)
Bell and Williams (2018)	Cross-over RCT	20	70.50 ± 5.49, (62, 81)	Haskap berry	100 mg of anthocyanins	Acute effect	Memory (Auditory Verbal Learning Task)	No significant differences found
							Executive functioning (Serial Subtraction)	Significant and positive effect as assessed by the serial subtraction test (7s errors)
							Attention and concentration (Attention Network Task)	No significant differences found
				Haskap berry	200 mg of anthocyanins	Acute effect	Memory (Auditory Verbal Learning Task)	No significant differences found
							Executive functioning (Serial Subtraction, 3s and 7s)	Significant and positive effect as assessed by the serial subtraction test (3s errors)
							Attention and concentration (Attention Network Test)	No significant differences found
				Haskap berry	400 mg of anthocyanins	Acute effect	Memory (Auditory Verbal Learning Task)	Significant and positive effect as assessed by the Auditory verbal learning task (recognition)
							Executive functioning (Serial Subtraction, 3s and 7s)	Significant and positive effect as assessed by the serial subtraction test (7s errors)
							Attention and concentration (Attention Network Test)	No significant differences found
Watson et al. (2018)	Cross-over RCT	9	23 (mean)	Blackcurrants, *Ribes nigrum L.*	96.96 mL ~ 515.7 mg of polyphenols	Acute effect	Attention and concentration (CogTrack™)	No significant differences found
							Processing speed (CogTrack™)	Significant and negative effect as assessed by the CogTrack™ test (Choice reaction time)
							Attention and concentration (CogTrack™)	No significant differences found
Dodd et al. (2019)	Cross-over RCT	18	68.72 ± 3.30, (62, 73)	Blueberry	30 g ~ 200 g fresh fruit ~ 578.82 mg of polyphenols	Acute effect	Executive functioning (Go-NoGo, Correct Reaction Time, Digit Symbol Substitution Test, Total Correct)	No significant differences found
							Processing speed (Stroop, Correct Reaction Time, Digit Switch, Switch Cost)	No significant differences found
							Attention and concentration (Continuous Performance Task, Commission Errors)	No significant differences found
							Memory (Random Word Generation, Total Correct, Three-Word Sets, Total Correct)	No significant differences found
Ahles et al. (2020)	Parallel RCT	90 mg Aronia: 34 150 mg Aronia: 35 (32 controls)	90 mg Aronia: 53 ± 1 150 mg Aronia: 53 ± 1	Aronia melanocarpa	90 mg ~ 16 mg anthocyanins 150 mg ~ 27 mg anthocyanins	24 weeks	Motor skills and construction (Grooved Pegboard Test)	Significant and positive effect as assessed by the Grooved pegboard test (dominant hand)
							Attention and concentration (Number Cross-Out Test)	No significant differences found
							Processing speed (Stroop Color and Word Test)	No significant differences found
Jackson et al. (2020)	Cross-over RCT	32	22.28 ± 4.27	Blueberry	2.49 g ~ 300 mg anthocyanins	Acute effect	Executive functioning (COMPASS)	No significant differences found
							Processing speed (COMPASS)	No significant differences found
Whyte et al. (2021)	Cross-over RCT	35	51 ± 8	Blueberry (wild)	25 g ~ 1 cup fresh fruit ~ 725 mg polyphenols	Acute effect	Memory (Auditory Verbal Learning Test)	Significant and positive effect as assessed by the Auditory verbal learning test (word rejection accuracy)
							Executive functioning (Modified Attention Network Task)	No significant differences found
García-Cordero (2022)	Parallel RCT	20	56.4 ± 4.14	Red berry + blackcurrants + raspberries + blueberries	1 tablespoon ~ 100 mg anthocyanins	12 weeks	Memory (Memory Summary, Working Memory Summary)	No significant differences found
							Processing speed/Attention and concentration	No significant differences found
		19	57.84 ± 6.76	Red berry + blackcurrants + raspberries + blueberries	1 tablespoon ~ 100 mg anthocyanins	12 weeks	Memory (Memory Summary, Working Memory Summary)	No significant differences found
							Processing speed/Attention and concentration (Processing Speed and Attention Summary)	No significant differences found

^1^ If not specified, there was no control group. ^2^ If not otherwise stated: mean ± SD, (MIN, MAX). RCT, randomized controlled trial; SD, standard deviation; MIN, minimum; MAX, maximum.

## Data Availability

Data sharing not applicable. No new data were created or analyzed in this study. Data sharing is not applicable to this article.

## Supplementary Materials

The following supporting information can be downloaded at: https://www.mdpi.com/article/10.3390/nu14142977/s1. Table S1: Report of study characteristics [23,24,25,26,27,28,29,30,31,32,33,34,35,36,37]; Figure S1: Summary plot; Figure S2: Traffic light [23,24,25,26,27,28,29,30,31,32,33,34,35,36,37].
